# Adherence determination using urine-tenofovir point-of-care testing and pharmacy refill records: A cross-sectional study

**DOI:** 10.1097/MD.0000000000036321

**Published:** 2023-11-24

**Authors:** Ebiere C. Herbertson, Cecile D. Lahiri, Olubusola A. Olugbake, Rebecca O. Soremekun, Matthew A. Spinelli, Monica Gandhi

**Affiliations:** a Nigerian Institute of Medical Research, Lagos, Nigeria; b Department of Medicine, Division of Infectious Diseases, Emory University School of Medicine, Atlanta, GA; c Department of Clinical Pharmacy and Biopharmacy, University of Lagos, Lagos, Nigeria; d University of California at San Francisco (UCSF), San Francisco, CA.

**Keywords:** antibody-based, antiretroviral therapy, immunoassay, pharmacy-refill record, Tenofovir, urine test

## Abstract

Pharmacy refill records (PRR), are an accessible strategy for estimating adherence in low- and middle-income countries (LMICs). However, the low-cost urine-tenofovir point-of-care test opens up the possibility of an objective metric of adherence that is scalable to LMICs. This study compared adherence to tenofovir-based regimens using urine-tenofovir point-of-care (POC) test with pharmacy refill records in a Nigerian population of HIV-positive persons. This was a cross-sectional study among 94 HIV-positive adults, which was conducted from June to August 2021, in a large outpatient clinic in Lagos, Nigeria. Adherence to pharmacy appointments was automatically calculated using a computerized pharmacy appointment system (FileMaker Pro™). Urine drops on the urine-tenofovir POC test strip developed 2 lines for a negative test (tenofovir absent) and one line for a positive test. Fisher’s exact test was used to examine the association between pharmacy refill record and urine-tenofovir point-of-care test. Logistic regression was performed to predict viral suppression (<1000 copies/mL, based on WHO recommendations) using both methods of adherence determination. A Receiver Operating Characteristic (ROC) curve of the association between specificity and sensitivity was generated to evaluate the predictive value of adherence determined using pharmacy-refill record and urine-tenofovir point-of-care test in forecasting viral suppression. The statistical significance level was set at 0.05. Fisher’s exact test showed no statistically significant difference in adherence using urine-tenofovir point-of-care test or pharmacy refill record. The logistic regression model showed that an increase in pharmacy-refill record of ≥ 95% was associated with viral suppression (*P* = .019). From the ROC curve, the sensitivity was same at 95.5% for both methods, but the specificity of the urine-tenofovir point-of-care test was greater (96.6% vs 95.5%) than pharmacy refill record (*P* = .837). Urine-tenofovir point-of-care test provided equivalent adherence data to pharmacy refill data.

## 1. Introduction

There were an estimated 39 million people globally living with the Human Immunodeficiency Virus (HIV) at the end of 2022.^[1]^ Sixty-five percent of these persons living with HIV (PLHIV), were from Sub-Saharan Africa.^[2]^ Nigeria, a West African Country with a population of 313.4 million people^[2]^ reported an HIV prevalence of 1.4% in 2018.^[3]^ Nigeria has the third largest population of PLHIV,^[2,4]^ and has also been referred to as “the largest HIV epidemic in Western and Central Africa.”^[5]^

The world is making good progress towards the targets that were set to end the HIV epidemic by 2030. Five Southern African countries were reported to have achieved the 95-95-95 targets in 2022.^[1]^ In order words, 95% of the population of these countries knew their HIV status, 95% of those who knew their HIV status were on life-saving antiretroviral therapy, and 95% of PLHIV who were on antiretroviral therapy, had suppressed HIV viral load results. The level of the USAID 95-95-95 targets in Nigeria in 2022 were 86:89:93 among adults aged 15-45 years old.^[2]^ Nigeria has adopted policies which “lead to the path that ends AIDS.”^[2]^ Some of these policies include adoption of the World Health Organisation recommendation of rapid initiation of antiretroviral therapy (ART) within 7 days of HIV diagnosis (Treat-all policy).^[2]^ The treat-all policy (regardless of CD4 count) is implemented in over 95% of all HIV treatment sites for adults and adolescents in the country.^[2]^ Other policies such as multi month dispensing, 6-monthly viral load monitoring (for stable patients) or 3-monthly for patients who have unsuppressed viral load results, are outlined in the National HIV Treatment Guidelines.^[3]^

The Nigerian National Treatment Guidelines recommend intensified adherence counseling for patients with unsuppressed viral load.^[6]^ Adherence is a patient’s ability to follow a treatment plan, take medications at prescribed times and frequencies, and follow restrictions regarding food and other medications.^[7]^ There are few antiretroviral drugs available for combination antiretroviral therapy options in low- and middle-income countries (LMIC). Ensuring proper adherence to available antiretroviral drugs is very important to avoiding the development of resistance to the few accessible antiretroviral drugs.

Adherence assessment is critical to the success of ART and pre-exposure prophylaxis (PrEP). Commonly used measures of adherence such as “self-report,” or “pharmacy-pick-up”/ “pharmacy refill records,” are acclaimed not to be as reliable as objective adherence tests measuring antiretroviral (ARV) concentrations in biological samples.^[8]^ Unlike most other currently available objective tests (measuring drug levels in plasma, hair, or red blood cells), which involve expensive and time intensive mass-spectrometry-based methods, antibody-based urine-tenofovir immunoassay which can be performed at the point-of-care (POC), is easy to conduct, and results are available in real-time (3–5 minutes).^[9]^ A point-of-care test allows for a rapid assessment of adherence levels in a clinic, as well as providing immediate feedback to an individual patient, which can help initiate intensive adherence counseling targeted to the patient.^[9]^ Point-of-care monitoring and feedback could improve adherence to ART and help increase viral suppression rates.

Concentrations of tenofovir in urine have been successfully used to monitor adherence to PrEP.^[9]^ Paired urine and plasma tenofovir concentrations have been shown to be highly correlated^[10]^ and measuring tenofovir levels in urine assesses adherence.^[11]^ Beyond the high predictive utility of an adequate urine-tenofovir level to assess adherence to PrEP, urine-tenofovir concentrations can evaluate time since dosing.^[12]^ Since tenofovir is the most widely used nucleotide reverse transcriptase inhibitor in HIV treatment and prevention, the University of California, San Francisco Hair Analytical Laboratory helped to develop a highly selective antibody against tenofovir which was converted into an enzyme-linked immunosorbent assay and validated via liquid chromatography/tandem mass spectrometry.^[12]^ A lateral flow assay, which permits POC testing, was then developed, which examines recent adherence over the last 4 to 7 days based on a cutoff of 1500 nanograms (ng)/milliliter (mL) of tenofovir. Absence of tenofovir detected by our assay predicted future PrEP seroconversion in large demonstration projects (iPrEx OLE study and Partners PrEP).^[13,14]^ Moreover, urine collection has been demonstrated to be highly acceptable to research participants and patients.^[12,15,16]^

The urine-tenofovir POC test will be very useful for special populations such as adolescents and young adults who are reported to have problems with adherence to ART^[17]^; and pregnant women, for whom adherence is very important to prevent mother-to-child transmission of HIV.

Since self-reported adherence and pharmacy-refill record are easy to assess in LMIC, these metrics are used most often to estimate population or individual level adherence. However, the low-cost urine tenofovir test opens up the possibility of an objective metric of adherence that is scalable to LMICs.

The purpose of this study was therefore to compare adherence to tenofovir-based antiretroviral regimens by the urine-tenofovir point-of-care test and pharmacy-refill record in a Nigerian population of people living with HIV, and to compare the HIV viral load suppression predictive ability of each measure of adherence.

## 2. Methods

### 2.1. Study setting

The Outpatient HIV Clinic in Lagos, Nigeria, is situated in the Nigerian Institute of Medical Research which is charged with conducting research relevant to the country. The Institute was among the 25 centers selected in 2002 to implement the Federal Government of Nigeria’s antiretroviral drug access programme. The Institute was selected principally to conduct clinical research in the large antiretroviral therapy (ART) access programme. Cumulatively over 24,000 HIV positive individuals have been enrolled into the HIV programme since its inception. A diverse population of persons living with HIV (PLHIV) access care at the Nigerian Institute of Medical Research clinic. The clinic population include infants exposed to HIV, children and adolescents, pregnant women, and other adults. This cross-sectional study to evaluate the relationship between urine-tenofovir test results and pharmacy refill records was conducted between June to August 2021.

### 2.2. Study design

This was a cross sectional study.

### 2.3. Ethical approval and informed consent

Ethical approval for the study was obtained from the Institutional Ethics Review Board of Nigerian Institute of Medical Research (IRB-16-360 of 21-06-2022). All study data was de-identified, confidentiality of participants and their data was maintained all through the study. Informed consent was obtained from study participants, refusal to participate in the studies, did not affect the quality of care a patient received at the clinic.

### 2.4. Inclusion criteria

HIV-positive adults (≥18 years old) on first-line or second-line tenofovir-based ART regimen.

### 2.5. Exclusion criteria

Patients on non-tenofovir-based ART regimens. This is because the urine-tenofovir test can only detect tenofovir metabolites and would not react to metabolites of other antiretroviral drugs.

Sample size calculation for the study was based on proportion of 27% of non-adherence reported for pharmacy refill records from 2 African studies.^[18,19]^ Assumptions made in the sample size calculation were, 5% precision, 5% type 1 error, and population size < 10,000. was The calculated sample size was ninety-one (91), and 100 persons were assessed for study participation eligibility. Four of those assessed were ineligible because they were not on tenofovir-based ART regimens while 2 persons declined providing consent. Ninety-four study participants who were PLWH on tenofovir disoproxyl fumerate-based regimens (on first- or second-line ART) were therefore recruited consecutively from the clinic and consented for participation.

The urine tenofovir immunoassay was donated by Hair Analytical Laboratory, San Francisco, and the following steps conducted for each participant:

Each participant was presented with a cup and asked to provide a urine sample (about 5–10 mL).

**Figure 1.**
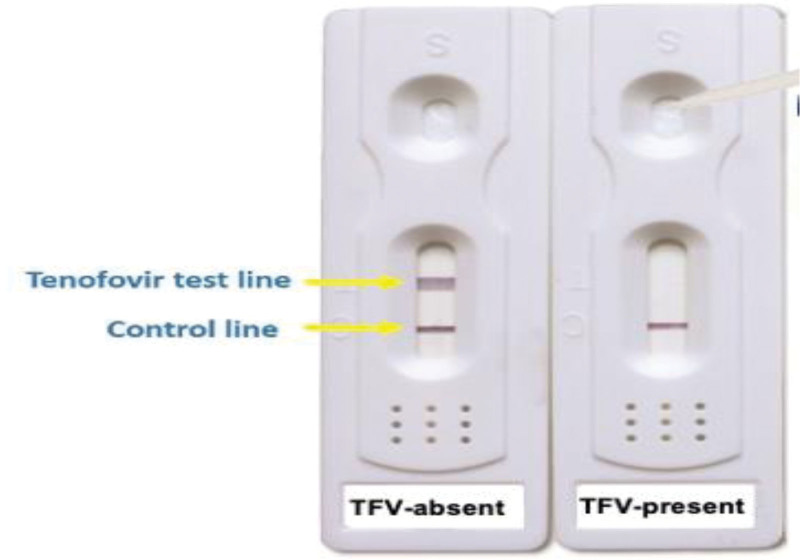
Urine-tenofovir POC test strip. POC = point of care.

1. They were asked to screw the lid on to the urine cup after collection and bring it back out to the researcher.
2. The researcher (using gloves) used the dropper provided with the urine test to drip 3 to 4 drops of urine from the urine cup into the divot of the urine test strip (indicated by blue arrow on the picture, Fig. 1).
3. After applying the urine drops to the urine strip test, the strip was laid on a paper towel and left to develop for 3 to 5 minutes.

Adherence determination using pharmacy pick-up records: Adherence using pharmacy pick-up record was calculated automatically by a computerized pharmacy appointment system (FileMaker Pro™ version 12.4, Cupertino, CA) which uses the total number of days that the patient was late/absent for drug-refill visits to calculate adherence to ART. Optimal and suboptimal adherence was defined as a cumulative adherence to drug-refill visits of ≥ 95% and < 95% respectively.

Statistical tests: International Business Machine (IBM) Statistical Package for Social Sciences (SPSS) version 26.0, Armonk, NY: IBM Corporation USA was used for statistical analysis. Statistical significance was set at 5% (0.05) level of probability. Fisher’s exact test was used to examine the association between pharmacy pick-up data (≥95% and < 95%) and the tenofovir urine assay (yes/no). A logistic regression was performed to predict viral suppression with adherence measured using the urine tenofovir POC test and pharmacy refill records. A receiver operating characteristic (ROC) curve of the association between specificity and sensitivity was generated to evaluate the predictive value of pharmacy refill records and the urine POC test, in forecasting viral suppression. The ROC curve was constructed by plotting the false positive rate (1 minus specificity) against the true positive rate (sensitivity). Detectable viral load was defined as greater than 1000 copies/mL.^[20]^ The adherence measure with the greatest total area was interpreted as having the best prediction of viral load suppression.

## 3. Results

A total of 94 participants were recruited for the study (Table 1) majority of whom 63(67%) were female. The age range of the study participants was 18 to 62 years and the mean age was 43.9 years (standard deviation ± 12.3). Forty-three out of the ninety-four (45.7%) of study participants were on first-line antiretroviral therapy while 51 (54.3%) were on second-line antiretroviral therapy. A large proportion of the study participants on first-line antiretroviral therapy 41 (95.3%), were on dolutegravir and tenofovir-based regimens. Boosted atazanavir was the most used Protease Inhibitor for second-line antiretroviral therapy 44 (86.3%). Most of the participants, 93% of those on first-line antiretroviral therapy and 94.1% of those on second-line antiretroviral therapy were virologically suppressed (HIV viral load < 1000 copies/mL).

**Table 1:** Demographic and baseline clinical characteristics of study participants.

Characteristic	First-line ART	Second-line ART	Total, n (%)
		Value	
Total no. of participants – n (%)	43 (45.7)	51 (54.3)	94 (100)
Female – n (%)	26 (60.5)	37 (72.5)	63 (67)
Male – n (%)	17 (39.5)	14 (27.5)	31 (33)
On LPV/r + TDF/3TC – n (%)	0	7 (13.7)	7 (7.4)
On ATV/r + TDF/3TC – n (%)	0	44 (86.3)	44 (46.8)
On DTG/ TDF/3TC – n (%)	41 (95.3)	0	41 (43.6)
On EFV/ TDF/3TC – n (%)	2 (4.7)	0	2 (2.1)
Viral load ≤ 1000 copies/L – n (%)	40 (93.0)	48 (94.1)	88 (93.6)
Viral load > 1000 copies/L – n (%)	3 (7.0)	3 (5.9)	6 (6.4)
Mean age -year ± (SD)	39.8 ± (13.0)	45 ± (8.2)	43.9 ± (12.32)

3TC = Lamivudine, ATV/r = Atazanavir (boosted with ritonavir), DTG = Dolutegravir, EFV = Efavirenz, LPV/r = Lopinavir (boosted with ritonavir), SD = Standard deviation.

The result of the Fisher’s exact test, which is shown in Table 2 demonstrates no statistically significant difference in adherence measured using either the urine-tenofovir point-of-care test or pharmacy refill records.

**Table 2:** Fisher’s exact test.

Adherent	Adherence determination method		Statistics
	Urine-Tenofovir POC	Pharmacy record	
Yes	88	89	Fisher’s exact
No	6 (6.38%)	5 (5.32%)	*P* = .201

The logistic regression which was performed to predict viral suppression with adherence measured using urine-tenofovir point-of-care test and pharmacy refill records, showed that the logistic regression model was statistically significant, χ^2^ (2) = 10.627, *P* = .005 (Table 3).

**Table 3:** Logistic regression to predict viral suppression with adherence measured using pharmacy pick-up record (PPR) and urine-tenofovir point-of-care test.

Variables in the equation	Statistics						95% CI for Exp(B)	
	B	S.E	Wald	df	Sig.	Exp(B)	Lower	Upper
TDF present	0.959	1.379	0.483	1	0.487	2.609	0.175	38.966
PPR ≥ 95%	-2.517	1.073	5.499	1	0.019	0.081	0.010	0.662
Constant	2.163	1.347	2.580	1	0.108	8.697		

TDF Present = Positive adherence from urine test, PPR ≥ 95% = Adherence equal or greater than 95% from pharmacy pick-up record, Constant = viral load.

The model explained 15.5% (Nagelkerke *R*^2^) of the variance in viral suppression and correctly classified 94.7% of cases. It also showed that an increase in pharmacy refill rate of ≥ 95% was associated with viral suppression (*P* = .019). The contribution of urine POC to the model was however not statistically significant (*P* = .487).

**Figure 2.**
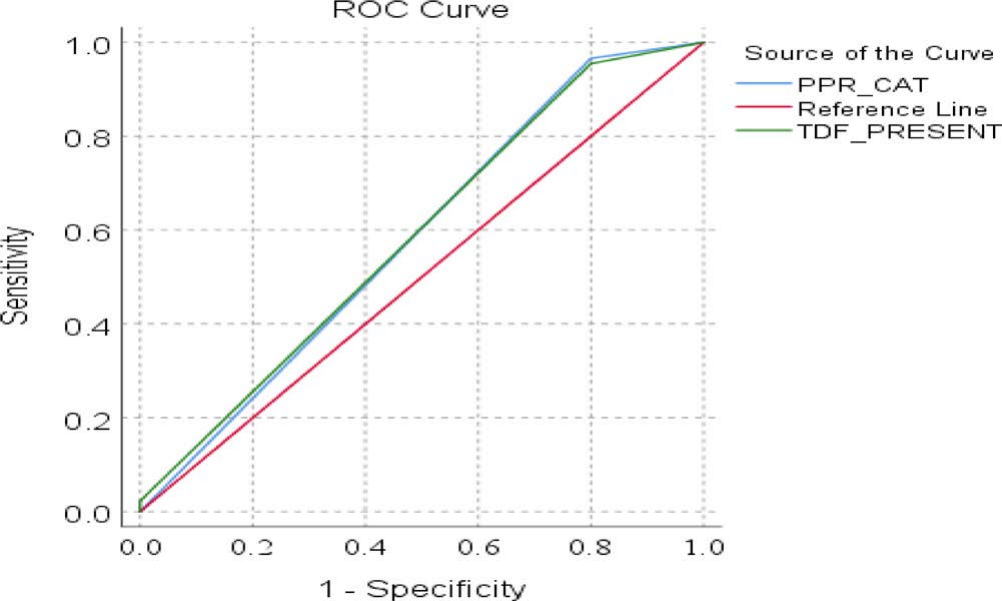
ROC comparing pharmacy pick-up record and urine-tenofovir POC test; black diagonal – reference, blue line – pharmacy pick-up record, green line – urine tenofovir POC test. POC = point of care, ROC = receiver operating characteristic curve.

Figure 2 shows the ROC curves of the association between specificity and sensitivity to compare the performance of both methods of adherence determination. Sensitivity was 95.5% for both methods (Table 4), but the sensitivity of the urine-tenofovir point-of care test was greater (96.6% vs 95.5%) than pharmacy refill record (*P* = .837).

**Table 4:** Area under the curve, sensitivity and specificity of both methods of adherence measurement (urine-tenofovir point-of-care test and pharmacy pick-up records).

	Urine-TNF POC	Pharmacy refill record	*P* value
ROC AUC	0.586	0.583	.837
Sensitivity (%)	96.6	95.5	
Specificity (%)	80.0	80.0	

AUC = area under the curve, POC = point of care, ROC = receiver operating characteristic curve (Figure 2).

## 4. Discussion

Our study showed that there was no difference between adherence measured using either pharmacy pick-up record or urine-tenofovir point-of-care test. It also showed that an increase in pharmacy refill rate of ≥ 95% was associated with viral suppression. Our data on pharmacy pick-up which is a good surrogate of adherence corroborates earlier reports of the high specificity of pharmacy pick-up records in identifying individuals who are not taking enough medication to attain treatment goals.^[21,22]^ Our findings are however at variance with some other previous studies which reported that pharmacy refill record was not a reliable method of adherence determination.^[23]^ Of note, this finding is valid only for closed-pharmacy systems^[24]^ where patients access medications from only one source, and the Nigerian National ARV programme is implemented as a closed-pharmacy system where patients can only pick-up ARVs from the pharmacy at the treatment center where they are registered.

In terms of the cutoff we used for the pharmacy pick-up record categorization, studies vary in the definition of the optimal level of adherence, using ≥ 85%, ≥ 90%, or ≥ 95% of pills taken as the threshold level necessary to achieve viral load suppression.^[25–28]^ A cutoff of 80% adherence has been demonstrated to successfully define viral suppression at both < 1000 copies/mL and 200 copies/mL.^[29]^ The level of adherence that is required to achieve HIV viral suppression is now no longer clear because with newer antiretroviral treatments, lower levels of adherence may still achieve successful virologic suppression.^[29,30]^ This work however adopted the most stringent adherence cutoff of 95% as defined by Paterson et al,^[25]^ and viral suppression was defined as < 1000 copies/mL as specified by the WHO 2016 Consolidated Guidelines for HIV^[20]^ and the Nigerian National HIV Treatment Guidelines.^[3]^

Urine-tenofovir POC test provided equivalent adherence data to pharmacy refill data. The performance of the urine tenofovir test opens up the possibility of an objective metric of adherence that is scalable to LMICs. Unlike most other currently available objective tests (measuring tenofovir levels in plasma, hair, or red blood cells) which cost about $140 to $200 per sample, urine-tenofovir POC is cheaper at $10 per test.

The main study limitation was its cross-sectional nature and future studies will examine this relationship over time. On the other hand, there was no bias introduced from the participants knowing their pharmacy refill data was being monitored by this one-time evaluation.

Another limitation of this study is the small sample size which may not allow for extrapolation of results. This result however provides preliminary data valid to “closed-pharmacy” settings, where patients receive ARV from only one source.

## 5. Conclusion

This study showed that the urine-tenofovir point-of-care test provided equivalent adherence data to pharmacy pick-up data in the closed pharmacy system. The result portends well for the utility of the inexpensive urine-tenofovir point-of-care test in low- and middle-income countries to objectively assess adherence.

## Acknowledgments

The authors gratefully acknowledge the Director-General, management staff, clinicians, pharmacists and data staff at Nigerian Institute of Medical Research. Mr. Samson Folami, a pharmacist on research internship, who assisted in conducting the urine-tenofovir POC test is also acknowledged.

## Author Contributions

Conceptualization: Ebiere Clara HERBERTSON

Formal analysis: Ebiere Clara HERBERTSON

Funding acquisition: Ebiere Clara HERBERTSON, Monica Gandhi

Investigation: Ebiere Clara HERBERTSON

Methodology: Ebiere Clara HERBERTSON, Matthew A. Spinelli

Writing—original draft: Ebiere Clara HERBERTSON

Supervision: Rebecca Soremekun, Olubusola Olugbake, Cecile Decille Lahiri, Monica Gandhi

Validation: Cecile Decille Lahiri, Monica Gandhi

Writing—review & editing: Cecile Decille Lahiri, Monica Gandhi

Visualization: Monica Gandhi
